# A rare case of tumor‐to‐tumor metastasis from thymic carcinoma to an ovarian mature teratoma

**DOI:** 10.1111/1759-7714.15277

**Published:** 2024-03-11

**Authors:** Tsuyoshi Sasada, Chigusa Shirakawa, Ryo Tachikawa, Daisuke Yamashita, Keisuke Tomii

**Affiliations:** ^1^ Department of Respiratory Medicine Kobe City Medical Center General Hospital Kobe Japan; ^2^ Department of Pathology Kobe City Medical Center General Hospital Kobe Japan

**Keywords:** distant metastasis, neoplasm metastasis, ovarian neoplasms, teratoma, thymus neoplasms

## Abstract

Metastasis from one neoplasm to another is referred to as tumor‐to‐tumor metastasis (TTM). TTM is rarely observed. Here, we present a patient with TTM from a thymic carcinoma to an ovarian mature teratoma. A 25‐year‐old woman, diagnosed with unresectable thymic carcinoma, presented with a cyst with a solid tumor component in her right ovary. Laparoscopic cystectomy of the right ovary revealed that the solid tumor was a distant metastasis of the thymic carcinoma in an ovarian mature teratoma. The possibility of malignant transformation of the ovarian mature teratoma was ruled out, enabling accurate staging of the thymic carcinoma. This case emphasizes the need for clinicians to consider TTM and the importance of pathological confirmation of TTM when investigating potential distant metastases.

## Graphical abstract

This is the first case of tumor‐to‐tumor metastasis (TTM) from a donor thymic carcinoma to a recipient ovarian mature teratoma. A 25‐year‐old woman who was diagnosed with unresectable thymic carcinoma presented with a cyst with a solid tumor component in her right ovary. Laparoscopic cystectomy of the right ovary revealed that the solid tumor was a distant metastasis of the thymic carcinoma in an ovarian mature teratoma. When investigating potential distant metastases, histopathological confirmation of TTM is beneficial.

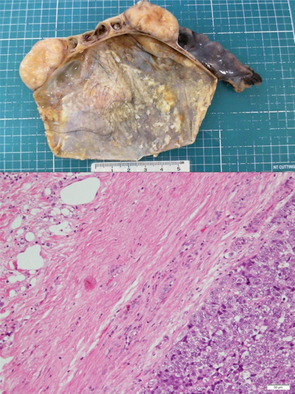

## INTRODUCTION

Tumor‐to‐tumor metastasis (TTM) is a hematogenous metastasis from one tumor (donor) to another (recipient) and is an extremely rare phenomenon. Approximately 150 cases of TTM have previously been reported in the literature.^1^ The most frequently reported donor tumors are lung and breast cancers, and the recipient tumors are predominantly clear cell renal carcinoma and meningioma.^1^, ^2^ Here, we present the first case of TTM in which a thymic carcinoma metastasized to an ovarian mature teratoma.

## CASE REPORT

**FIGURE 1:**
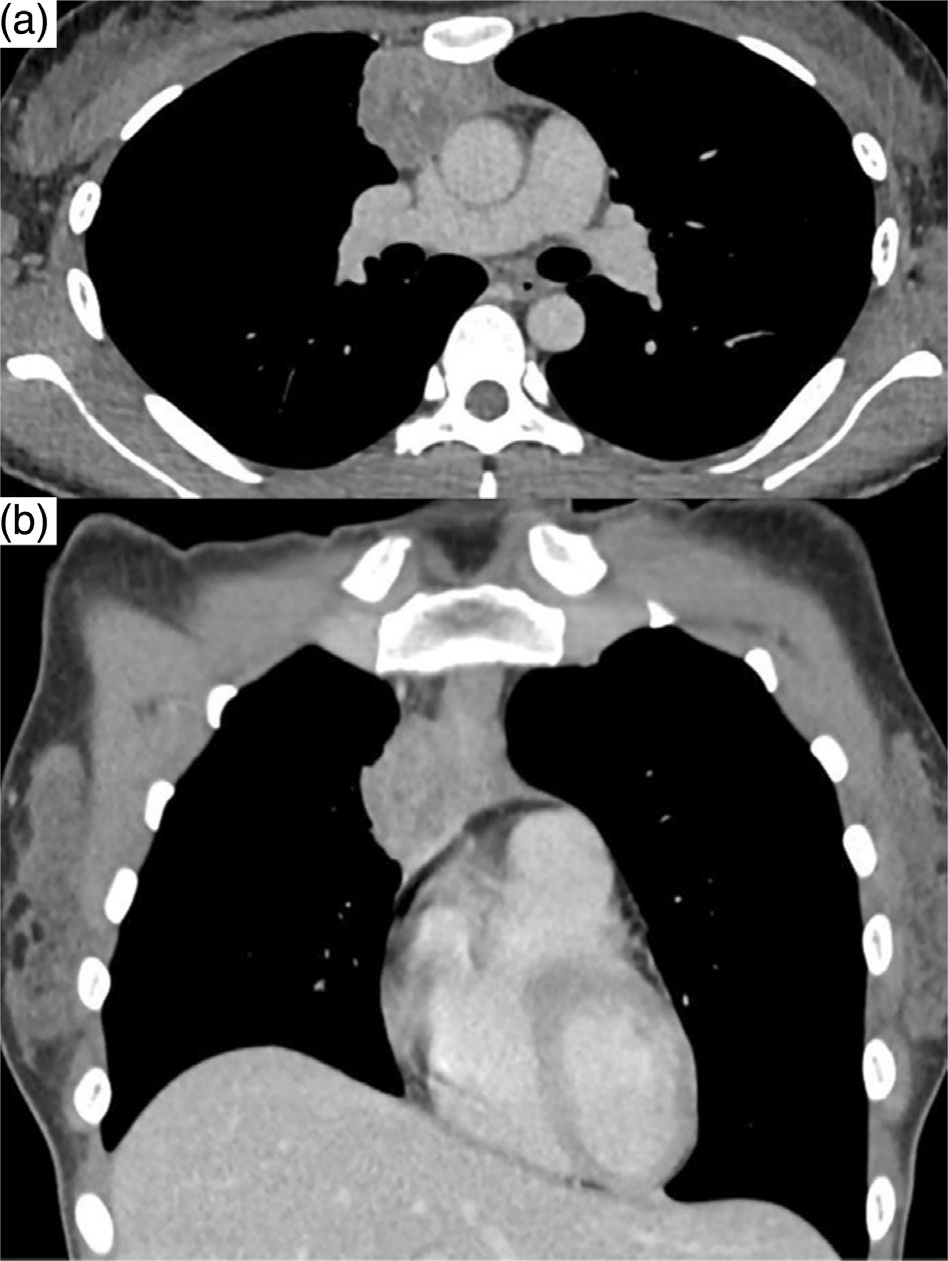
Contrast‐enhanced computed tomography of the thorax on initial examination. (a) Cross‐sectional and (b) coronal thoracic images.

**FIGURE 2:**
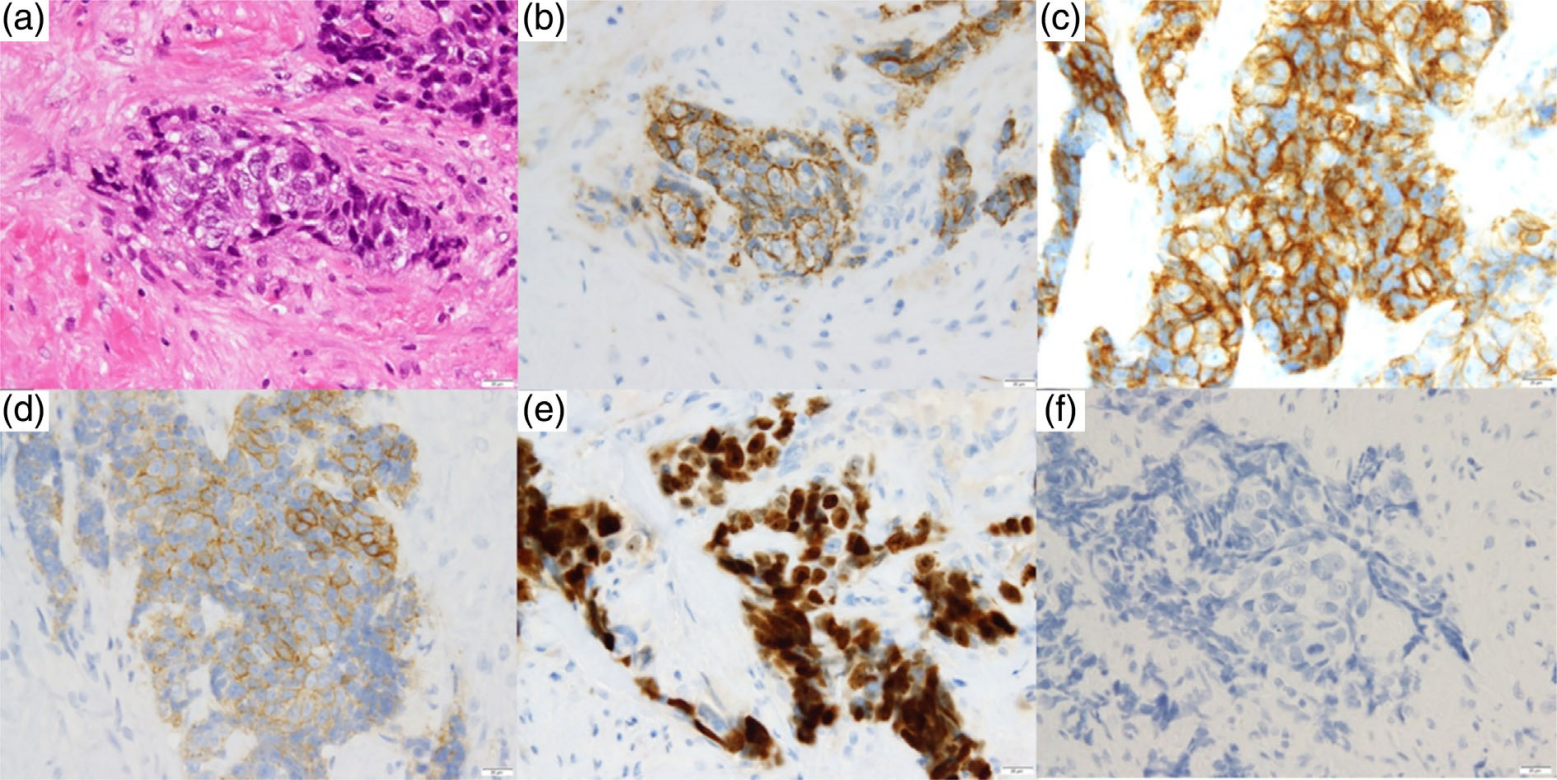
Pathological findings of surgical biopsy specimens. (a) Hematoxylin and eosin stain, (b) CK AE1/AE3 stain, (c) CD5 stain, (d) c‐kit stain, (e) Ki67 stain, and (f) sall4 stain images at 40x magnification.

**FIGURE 3:**
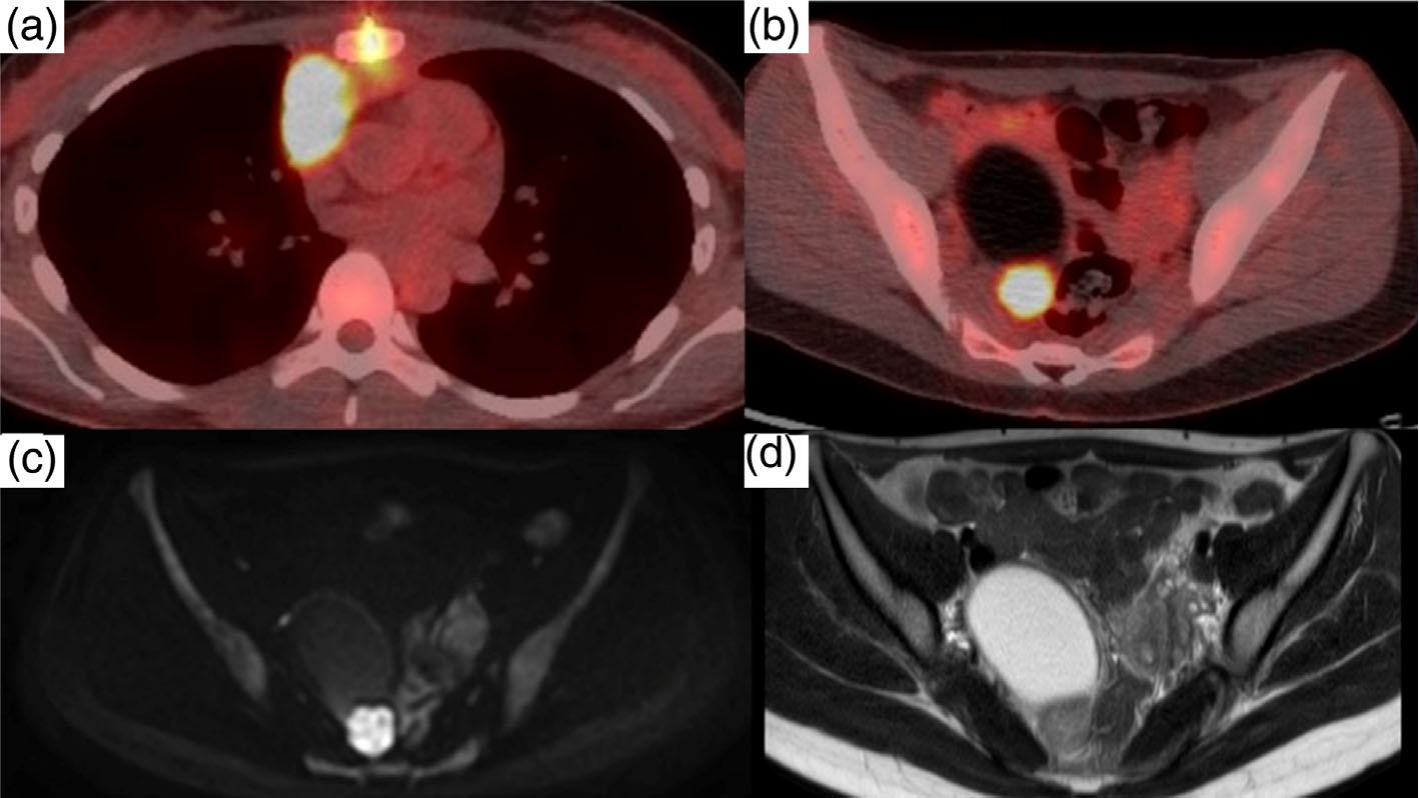
(a) Fluorodeoxyglucose‐positron emission tomography of the thorax in a horizontal section. (b) Fluorodeoxyglucose‐positron emission tomography of the pelvic region in a horizontal section. (c) Diffusion‐weighted magnetic resonance imaging of the right ovarian cyst in a horizontal section. (d) T2‐weighted magnetic resonance imaging of the right ovarian cyst in a horizontal section.

**FIGURE 4:**
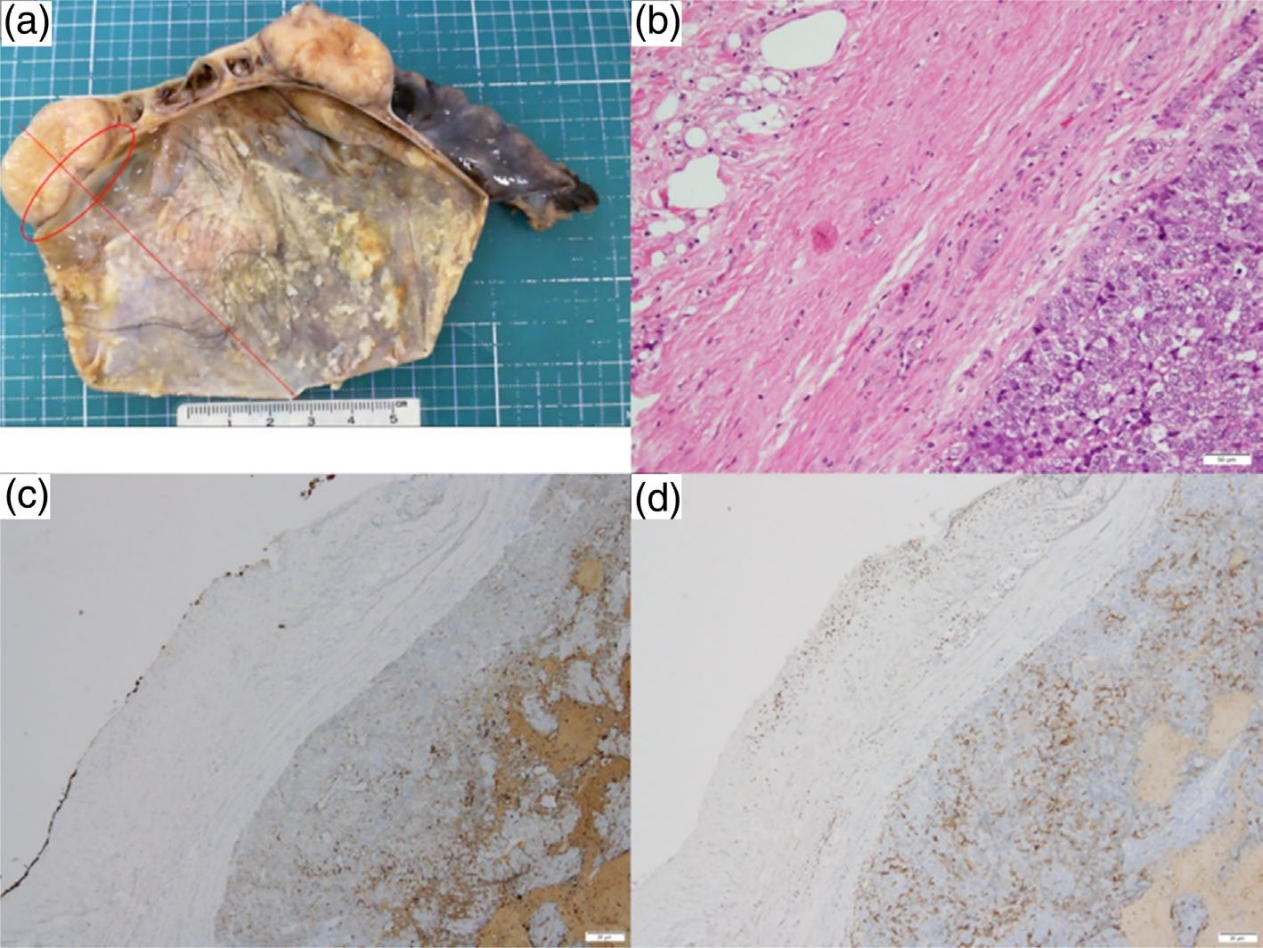
(a) The right ovary specimen, removed by laparoscopic right ovarian cystectomy, shows the split plane placed along the red straight line; the red oval area was sampled. (b) Epithelial tumor cells on the right invade the adipose tissues on the left (hematoxylin and eosin stain, 10x magnification). (c) Positive CK AE1/AE3 stain of the epithelial tumor cells (40x magnification). (d) Positive CD5 stain of the epithelial tumor cells (40x magnification).

A 25‐year‐old, non‐smoking woman, with no previously identified medical history presented to our outpatient clinic. Although the patient was asymptomatic, chest radiography revealed an enlarged right pulmonary hilar region. Blood test results, including those for specific tumor markers for lung cancer and thymic epithelial tumors, were within the normal ranges. Contrast‐enhanced computed tomography revealed a 44‐mm mass in the right anterior mediastinum with irregular borders and heterogeneous enhancement and pleural invasion (Figure 1a,b). During surgical evaluation, tumor seeding was observed in the pericardium, rendering the tumor unresectable. Histological examination revealed the proliferation of spindle‐shaped dysplastic epithelial cells (Figure 2a) that tested positive for CK AE1/AE3, CD5, c‐kit, Ki67, and sall4, confirming the diagnosis of thymic carcinoma (Figure 2b–f). Postoperative fluorodeoxyglucose‐positron emission tomography revealed a mass in the right ovary and the known thymic carcinoma in the anterior mediastinum (Figure 3a,b). Magnetic resonance imaging further characterized the ovarian lesion as consisting of both cystic and solid components (Figure 3c,d). The differential diagnoses included thymic carcinoma metastasis, malignant transformation of an ovarian mature teratoma, or another primary malignancy. Laparoscopic cystectomy of the right ovary revealed hair and fat tissues within the cystic portion, consistent with ovarian mature teratoma (Figure 4a). Histological examination indicated the infiltration of epithelial dysplastic cells from the solid section into adipose tissues from the cystic section (Figure 4b). Immunohistochemical staining of the solid section confirmed markers characteristic of thymic carcinoma, including CK AE1/AE3 and CD5 (Figure 4cd). Therefore, the patient was diagnosed with TTM of thymic carcinoma to an ovarian mature teratoma. The patient was staged as IVB (T4N0M1b), and chemotherapy was initiated.

## DISCUSSION

TTMs are commonly observed as metastases from poorly differentiated tumors to highly differentiated tumors, which are characterized by hypervascularity and higher fat and sugar contents.^3^ This phenomenon aligns with Paget's seed and soil theory.^3^, ^4^ Therefore, ovarian mature teratomas, known for their high‐fat content and hypervascularization, represent an ideal soil environment for TTM. The four established criteria for TTM postulated by Campbell in 1968^3^ were met in this patient: (1) presence of two or more primary tumors, (2) disorganized and dependent donor and recipient tumors, (3) metastatic spread to remote sites rather than through continuous infiltration or tumor thrombus, and (4) absence of any infiltration into the lymphatic system causing distant metastasis. In the present patient, pathological evaluation of the right ovary successfully ruled out malignant transformation within the ovarian mature teratoma, confirming TTM from thymic carcinoma.

Thymic carcinoma is an extremely rare tumor type, and distant metastasis from thymic carcinoma is uncommon. Distant metastasis is observed in 1.2% of patients with thymomas and 12.0% of patients with thymic carcinoma.^5^ The most frequent sites of metastases are the lungs, bones, and liver. Although no report has described TTM from a thymic epithelial tumor to an ovarian tumor, highly malignant thymic epithelial tumors presenting with ovarian metastases have been reported in previous studies.^6^, ^7^, ^8^ Case reports documenting metastases to the brain, pancreas, and small intestine have also been reported.^9^, ^10^, ^11^

Although there have been no reports of TTM in which thymic carcinoma was the donor, TTM involving ovarian tumors as recipients of various primary tumors has been reported in 16 previous cases.^12^, ^13^, ^14^ The primary sources of these metastases were predominantly breast and cervical cancers. Focusing only on cases in which ovarian mature teratomas were the recipients, the donor tumors were appendiceal cancer, cervical adenocarcinoma, and breast cancer. As in the current report, histopathological examination was essential to distinguish between TTM and malignant transformation of ovarian mature teratoma in the previous cases.^12^, ^15^, ^16^

The pathogenesis of metastatic spread from thymic carcinoma to mature ovarian teratomas is not fully understood. The cell adhesion molecule claudin‐4 has been implicated in this process.^17^ Claudin‐4 is highly expressed in the medullary portion of the thymus and has been identified in both thymomas and thymic carcinoma.^18^ Claudin‐4 is overexpressed in ovarian epithelial cells and ovarian tumors.^19^ Thymic carcinoma cells exhibiting mesenchymal characteristics in the bloodstream are required to undergo epithelial transition for successful colonization at the metastatic site. Elevated levels of Claudin‐4 may facilitate this colonization process at the metastatic site.^18^

To the best of our knowledge, this is the first documented case of TTM with thymic carcinoma as a donor tumor to an ovarian mature teratoma. Clinicians should consider the possibility of TTM from poorly‐differentiated epithelial tumors to well‐differentiated, highly vascularized, and nutrient‐rich tumors to ensure accurate staging and assessment of disease progression. If TTM is suspected, a diagnosis must be determined using histopathological examination.

## AUTHOR CONTRIBUTIONS

All authors had full access to the data in the study and take responsibility for the integrity of the data and the accuracy of the data analysis. Tsuyoshi Sasada: Writing of the first draft, investigation and visualization. Chigusa Shirakawa: Conceptualization, writing of the first draft, investigation and supervision. Ryo Tachikawa: Conceptualization, supervision, manuscript review and editing. Daisuke Yamashita: Preparation and imaging of pathology specimens, investigation and supervision. Keisuke Tomii: Manuscript review and editing and project administration.

## FUNDING INFORMATION

This study did not receive any specific grants from funding agencies in the public, commercial, or not‐for‐profit sectors.

## CONFLICT OF INTEREST STATEMENT

The authors declare no conflict of interest.

## Data Availability

No additional data are available for this study.

## References

[1] Minezaki S , Misawa T , Tsukayama H , Shibuya M , Wada K , Sano K , et al. Tumor‐to‐tumor metastasis: an extremely rare combination with renal cell carcinoma as the donor and a pancreatic neuroendocrine tumor as the recipient. Surg Case Rep. 2022;8:8. 10.1186/s40792-022-01361-5 35001202 PMC8743331

[2] Cenkowski M , Gibson IW , Lategan B , Czaykowski PM . Tumor‐to‐tumor metastasis: report of a case of renal cell carcinoma metastasizing to a pancreatic endocrine neoplasm. J Clin Oncol. 2011;29:e303–e304. 10.1200/JCO.2010.33.2536 21245422

[3] Campbell LV Jr , Gilbert E , Chamberlain CR Jr , Watne AL . Metastases of cancer to cancer. Cancer. 1968;22:635–643. 10.1002/1097-0142(196809)22:3<635::AID-CNCR2820220320>3.0.CO;2-O 5673241

[4] Sawada T , Takahashi H , Hasatani K , Yoshida I , Oyama O , Inoue R , et al. Tumor‐to‐tumor metastasis: report of an autopsy case of lung adenocarcinoma metastasizing to renal cell carcinoma. Intern Med. 2009;48:1525–1529. 10.2169/internalmedicine.48.2176 19721297

[5] Kondo K , Monden Y . Lymphogenous and hematogenous metastasis of thymic epithelial tumors. Ann Thorac Surg. 2003;76:1859–1864; discussion 1864‐5. 10.1016/S0003-4975(03)01017-8 14667600

[6] Briese V , Rohde E . Ovarian metastasis of a thymoma. Zentralbl Gynakol. 1984;106:473–476. 6730770

[7] Martín‐Hernández R , Villanueva MM , Sánchez MN , López EC . Ovarian metastasis of a thymoma: report of a case and literature review. Int J Gynecol Pathol. 2015;34:374–378. 10.1097/PGP.0000000000000168 26061071

[8] Demirkiran F , Bese T , Arvas M , Yilmaz O , Ilvan S . Ovarian metastasis from malignant thymoma. Int J Gynaecol Obstet. 2009;105:176–177. 10.1016/j.ijgo.2008.12.005 19232597

[9] Kouitcheu R , Appay R , Diallo M , Troude L , Melot A . A case of brain metastasis of a thymic carcinoma with a review of the literature. Neurochirurgie. 2019;65:43–48. 10.1016/j.neuchi.2018.09.004 30711259

[10] Tsai YH , Lin KH , Huang TW . Rare solitary splenic metastasis from a thymic carcinoma detected on fluorodeoxyglucose‐positron emission tomography: a case report. World J Clin Cases. 2022;10:5072–5076. 10.12998/wjcc.v10.i15.5072 35801052 PMC9198860

[11] Yuan Y , Pu H , Pang MH , Liu YS , Li H . Thymic carcinoma metastasize to the small intestine: a case report. BMC Gastroenterol. 2020;20:358. 10.1186/s12876-020-01505-7 33115438 PMC7594467

[12] Yano M , Katoh T , Hamaguchi T , Kozawa E , Hamada M , Nagata K , et al. Tumor‐to‐tumor metastasis from appendiceal adenocarcinoma to an ovarian mature teratoma, mimicking malignant transformation of a teratoma: a case report. Diagn Pathol. 2019;14:88. 10.1186/s13000-019-0865-6 31409389 PMC6692929

[13] Fahoum I , Brazowski E , Hershkovitz D , Aizic A . Tumor‐to‐tumor metastasis of colorectal adenocarcinoma to ovarian cystadenofibroma. A case report and review of the literature. Int J Gynecol Pathol. 2020;39:270–272. 10.1097/PGP.0000000000000592 30882401

[14] Dundar B , Alrwashdeh A , Dahmoush L . Tumor to tumor metastasis: a case report of metastatic angiosarcoma to an ovarian Brenner tumor and review of the literature. Int J Gynecol Pathol. 2023;42:176–181. 10.1097/PGP.0000000000000854 35283447

[15] Santos F , Oliveira C , Caldeira JP , Coelho A , Félix A . Metastatic endocervical adenocarcinoma in a mature cystic teratoma: a case of a tumor‐to‐tumor metastasis. Int J Gynecol Pathol. 2018;37:559–563. 10.1097/PGP.0000000000000457 29140879

[16] Kirova YM , Feuilhade F , de Baecque‐Fontaine C , Le Bourgeois JP . Metastasis of a breast carcinoma in a mature teratoma of the ovary. Eur J Gynaecol Oncol. 1999;20:223–225. 10410892

[17] Li H , Ren B , Yu S , Gao H , Sun PL . The clinicopathological significance of thymic epithelial markers expression in thymoma and thymic carcinoma. BMC Cancer. 2023;23:161. 10.1186/s12885-023-10619-6 36797681 PMC9936685

[18] Martín de la Fuente L , Malander S , Hartman L , Jönsson JM , Ebbesson A , Nilbert M , et al. Claudin‐4 expression is associated with survival in ovarian cancer but not with chemotherapy response. Int J Gynecol Pathol. 2018;37:101–109. 10.1097/PGP.0000000000000394 28481779 PMC5815640

[19] Soini Y , Talvensaari‐Mattila A . Expression of claudins 1, 4, 5, and 7 in ovarian tumors of diverse types. Int J Gyencol Pathol. 2006;25:330–335. 10.1097/01.pgp.0000215298.38114.cc 16990707

